# Robust estimation of the expected survival probabilities from high-dimensional Cox models with biomarker-by-treatment interactions in randomized clinical trials

**DOI:** 10.1186/s12874-017-0354-0

**Published:** 2017-05-22

**Authors:** Nils Ternès, Federico Rotolo, Stefan Michiels

**Affiliations:** 1 Service de Biostatistique et d’Epidémiologie, Gustave Roussy, B2M, RdC.114 rue Edouard-Vaillant, 94805 Villejuif, France; 2 CESP, Fac. de médecine - Univ. Paris-Sud, Fac. de médecine - UVSQ, INSERM, Université Paris-Saclay, Villejuif, 94805 France

**Keywords:** Cox model, Penalized regression, Prediction model, Treatment-effect modifiers, Prognostic biomarkers, Survival estimation, Confidence intervals, Precision medicine, High-dimensional data

## Abstract

**Background:**

Thanks to the advances in genomics and targeted treatments, more and more prediction models based on biomarkers are being developed to predict potential benefit from treatments in a randomized clinical trial. Despite the methodological framework for the development and validation of prediction models in a high-dimensional setting is getting more and more established, no clear guidance exists yet on how to estimate expected survival probabilities in a penalized model with biomarker-by-treatment interactions.

**Methods:**

Based on a parsimonious biomarker selection in a penalized high-dimensional Cox model (lasso or adaptive lasso), we propose a unified framework to: estimate internally the predictive accuracy metrics of the developed model (using double cross-validation); estimate the individual survival probabilities at a given timepoint; construct confidence intervals thereof (analytical or bootstrap); and visualize them graphically (pointwise or smoothed with spline). We compared these strategies through a simulation study covering scenarios with or without biomarker effects. We applied the strategies to a large randomized phase III clinical trial that evaluated the effect of adding trastuzumab to chemotherapy in 1574 early breast cancer patients, for which the expression of 462 genes was measured.

**Results:**

In our simulations, penalized regression models using the adaptive lasso estimated the survival probability of new patients with low bias and standard error; bootstrapped confidence intervals had empirical coverage probability close to the nominal level across very different scenarios. The double cross-validation performed on the training data set closely mimicked the predictive accuracy of the selected models in external validation data. We also propose a useful visual representation of the expected survival probabilities using splines. In the breast cancer trial, the adaptive lasso penalty selected a prediction model with 4 clinical covariates, the main effects of 98 biomarkers and 24 biomarker-by-treatment interactions, but there was high variability of the expected survival probabilities, with very large confidence intervals.

**Conclusion:**

Based on our simulations, we propose a unified framework for: developing a prediction model with biomarker-by-treatment interactions in a high-dimensional setting and validating it in absence of external data; accurately estimating the expected survival probability of future patients with associated confidence intervals; and graphically visualizing the developed prediction model. All the methods are implemented in the R package biospear, publicly available on the CRAN.

**Electronic supplementary material:**

The online version of this article (doi:10.1186/s12874-017-0354-0) contains supplementary material, which is available to authorized users.

## Background

Thanks to the advances in genomics and targeted treatments, an increasing interest is being devoted to develop prediction models with biomarkers, called treatment-effect modifiers (for which the relative treatment effect varies according to the biomarker values), to predict how much benefit individual patients would derive from specific treatments. This aims at taking the therapeutic decision that fits the best each individual patient. Recently, there have been some attempts to identify prediction models that are associated with higher efficacy of particular treatments such as an 8-gene and a 14-gene signatures (i.e. prediction model) to evaluate the degree of trastuzumab benefit in early breast cancer patients on disease-free survival [1, 2]. Statistical methodology has been proposed for the development of prediction models and their validation [3, 4] and to estimate individual predictions in a survival setting [5, 6]; however, no clear guidance has been yet reached and evaluated in a high-dimensional setting.

Results from randomized clinical trials are often difficult to translate into predictions for individual patients, but the estimated absolute risk reductions from large randomized trials do still provide the best guidance [7]. Therefore, in addition to the challenges coming from the identification of treatment-effect modifiers in a high-dimensional setting, it is also important to identify prognostic biomarkers (i.e. associated with the clinical outcome and independently of treatment) to adjust for the main effect of established clinical and genomic variables in order to obtain individual survival probabilities. Indeed, the clinical impact of a treatment can be judged only with the knowledge of the prognosis of a patient. It is thus of importance to reliably predict the prognosis of patients to assist treatment counseling [8].

The aim of the present study was to propose a unified framework for developing and validating a high-dimensional Cox model [9] in a randomized clinical trial to estimate the expected treatment effect according to different values of the biomarkers included in the model, and to estimate survival probabilities for individual patients with associated confidence intervals. We propose different approaches that can be used in this context. We present a simulation study including null (i.e. with no treatment-effect modifier) and alternative scenarios (i.e. with at least one treatment-effect modifier) to evaluate the performance of the proposed approaches. We also illustrate the methods in a large randomized clinical trial of breast cancer patients. Finally, we discuss the findings.

## Methods

**Fig. 1:**
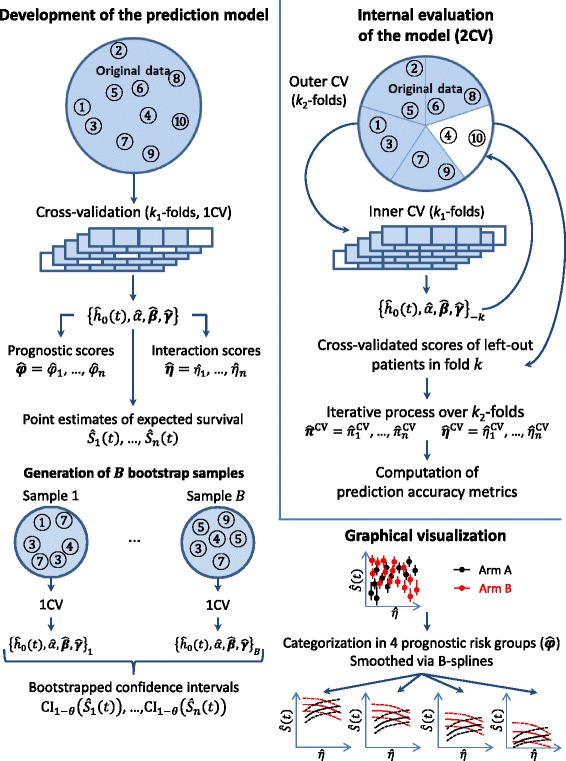
Schematic representation of the proposed framework. 1CV and 2CV: single and double cross-validation, *ĥ*_0_(*t*) baseline hazard at time *t*, $\widehat{\alpha},\widehat{\boldsymbol{\beta}},\widehat{\boldsymbol{\gamma}}$: estimated regression parameters, ${\widehat{\varphi}}_i = {\displaystyle \sum_{j=1}^p}{\widehat{\beta}}_j{X}_{i j}$: prognostic score, ${\widehat{\eta}}_i = {\displaystyle \sum_{j=1}^p}{\widehat{\gamma}}_j{X}_{i j}$: treatment-effect modifying score, $\widehat{\boldsymbol{\pi}}$: entire linear predictor of the selected model, iBrier: integrated Brier score, C: Uno’s C-statistic, ΔC: different in arm-specific Uno’s C-statistics, *Ŝ*(*t*): estimated survival probability at time *t*

In the present section we investigate several approaches to: (i) identify a parsimonious Cox regression model in a high-dimensional setting, (ii) estimate internally the predictive accuracy metrics of the selected model, (iii) estimate the expected survival probability for patients, (iv) construct confidence intervals thereof, and (v) visualize them graphically. A schematic representation of the framework is provided in Fig. 1.

### Identification of a prediction model with treatment-effect modifiers

The objective of developing a prediction model is to identify a model allowing the computation of a prognostic and a treatment-effect modifying score for each patient. From a statistical viewpoint, Rothwell put forward that the most reliable statistical approach for identifying treatment-effect modifiers is to test the interactions between the biomarkers and the treatment effect [7]. Hereinafter, we consider the proportional hazards model

1 $$ \begin{array}{l} h\left( t,\  T,\ \boldsymbol{X}\right)={h}_0(t) \exp \left(\alpha T + {\displaystyle \sum_{j=1}^p}{\beta}_j{X}_j + {\displaystyle \sum_{j=1}^p}{\gamma}_j{X}_j T\right)\\ {}={h}_0(t) \exp \left(\alpha T+{\boldsymbol{\beta}}^{\hbox{'}}\boldsymbol{X}+{\boldsymbol{\gamma}}^{\hbox{'}}\boldsymbol{X} T\right),\end{array} $$

where *α*, ***β*** = (*β*_1_, …, *β*_*p*_)^T^ and ***γ*** = (*γ*_1_, …, *γ*_*p*_)^T^ are the regression coefficients for: the treatment *T* coded as +0.5 and −0.5 for the experimental and control arm, respectively; the main effects of the *p* standardized biomarkers ***X*** = (*X*_1_, …, *X*_*p*_)^T^; and the *p* biomarker-by-treatment interactions, i.e. the product between the standardized biomarkers ***X*** and the treatment *T*. In other words, the first sum in (1) corresponds to an average prognostic component to the total score and the second sum in (1), corresponds to the treatment-effect modifying component. For simplicity, we do not consider clinical covariates in the presentation of the framework.

To overcome the nonidentifiability of the models due to the high-dimensional setting, we previously reviewed and proposed several methods to identify a parsimonious model starting from a large number of candidate biomarkers in a randomized clinical trial and to predict the magnitude of the relative treatment effect for future patients [10]. Most of these methods are based on penalized regression, which maximizes the penalized partial log-likelihood *l*_*p*_(*α*, ***β***, ***γ***, *λ*, *T*,***X***) that is the partial log-likelihood *l*(*α*, ***β***, ***γ***, *T*, ***X***) of model (1) minus a penalty:

$$ {l}_p\left(\alpha, \boldsymbol{\beta}, \boldsymbol{\gamma}, \lambda, T,\boldsymbol{X}\right)= l\left(\alpha, \boldsymbol{\beta}, \boldsymbol{\gamma}, \lambda, T,\boldsymbol{X}\right)-\lambda \left({\displaystyle \sum_{j=1}^p}{\theta}_j\left|{\beta}_j\right|+{\displaystyle \sum_{j=1}^p}{\vartheta}_j\left|{\gamma}_j\right|\right). $$

In the present work, we implemented two penalties: the lasso [11] which corresponds to *θ*_*j*_ = *ϑ*_*j*_ = 1, and the adaptive lasso [12, 13] which corresponds to ${\theta}_j=1/\left|{\widehat{\beta}}_j^{\mathrm{R}}\right|$, ${\vartheta}_j=1/\left|{\widehat{\gamma}}_j^{\mathrm{R}}\right|$, with ${\widehat{\beta}}_j^{\mathrm{R}}$ and ${\widehat{\gamma}}_j^{\mathrm{R}}$ the regression coefficients estimated in a preliminary step through the model (1) subject to the ridge penalty. As compared to the popular lasso penalty, the adaptive lasso penalty was initially proposed as it fulfilled the oracle property [14] in the prognostic setting. However, the performance of the oracle property is less known in high-dimensional samples with relatively low number of events. In a time-to-event setting with many candidate biomarker-by-treatment interactions, the adaptive lasso penalty was one of the best performing methods for selecting these interactions in a simulation study in [10]. For both the lasso and adaptive lasso, the tuning parameter *λ* was determined via a *k*_1_-fold cross-validation scheme (called 1CV for the rest of the article) by maximizing the cross-validated log-likelihood criterion of the Cox model [15]:

$$ c v l\left(\lambda \right)={\displaystyle \sum_{k=1}^{k_1}}\left({l}_p\left({\widehat{\alpha}}_{- k},{\widehat{\beta}}_{- k},{\widehat{\boldsymbol{\upgamma}}}_{- k},{\lambda}_{- k}, T,\ \boldsymbol{X}\right)-{l}_p\left({\widehat{\alpha}}_{- k},{\widehat{\beta}}_{- k},{\widehat{\boldsymbol{\upgamma}}}_{- k},{\lambda}_{- k},{T}_{- k},\ {\boldsymbol{X}}_{- k}\right)\right), $$

with *k*_1_ = 5 and where ${\widehat{\alpha}}_{- k}$, ${\widehat{\boldsymbol{\beta}}}_{- k}$, ${\widehat{\boldsymbol{\gamma}}}_{- k}$ and the tuning parameter are estimated in the training data excluding the subsample *k*: *T*_− *k*_ and ***X***_− *k*_. Penalized regression coefficients are estimated through the R-package glmnet [16, 17]. We also investigated the “refit adaptive lasso” and the “refit lasso” for which the selected non-zero regression coefficients are re-estimated in an unpenalized Cox regression model.

### Internal assessment of predictive accuracy of the prediction model

To assess the performance of a prediction model, some predictive accuracy measures (see the section “Simulation study” for more details) can be used. However, computing these metrics on the same data used to develop the model could lead to over-optimistic predictive accuracy measures and thus, external validation is crucial [18]. In the absence of external validation data set, a strategy suggested by Simon et al. [19] and illustrated by Matsui et al. [3] consists in performing a double cross-validation (2CV) to mimic an external validation. Indeed, patients are first divided into *k*_2_ subsamples (in this work *k*_2_ = 5); then, for each fold, the data in the fold are put apart and the data in the remaining *k*_2_ − 1 folds are used to estimate the tuning parameter *λ* through *k*_1_-fold cross validation and to estimate the prediction scores of the left-out patients. The process is iterated for all the *k*_2_ folds to estimate the cross-validated prediction scores of the complete data set. Of note, these scores obtained using the 2CV are only used to compute these prediction accuracy metrics, whereas the 2CV is not used elsewhere in this study.

### Point estimates of the expected survival probability

From the estimated model (1), the estimation of the expected survival probability of each patient *i* = (1, …, *n*) at time *t* can be obtained by plugging the estimated parameters in the survival function:

$$ {\widehat{S}}_i(t)=\widehat{S}\left( t,\ {T}_i,\ {\boldsymbol{X}}_{\boldsymbol{i}}\right)= \exp \left(-{\widehat{H}}_0(t)\times \exp \left(\widehat{\alpha}{T}_i + {\displaystyle \sum_{j=1}^p}{\widehat{\beta}}_j{X}_{i j} + {\displaystyle \sum_{j=1}^p}{\widehat{\gamma}}_j{X}_{i j}{T}_i\right)\right), $$

with *Ĥ*_0_(*t*) the cumulative baseline hazard at time *t*, that is commonly estimated through the non-parametric Breslow estimator [20].

### Estimation of confidence intervals of the expected survival probability

We compared two strategies to estimate the confidence intervals at level 1 – *θ* of the expected survival probabilities: one based on analytical expressions deriving from the normal approximation of the estimator and one based on a non-parametric bootstrap approach.

The analytical approach [21] consists in estimating the variance of the cumulative risk *Ĥ*_*i*_(*t*) = *Ĥ*(*t*, *T*_*i*_, ***X***_***i***_) based on the Breslow estimator [22] in the Cox model. Thus, the confidence interval of *Ŝ*_*i*_(*t*) is approximated as

$$ {\mathrm{CI}}_{1-\theta}\left({\widehat{S}}_i(t)\right)=\left[{q}_{\frac{\theta}{2}}\left({\widehat{S}}_i(t)\right);\ {q}_{1-\frac{\theta}{2}}\left({\widehat{S}}_i(t)\right)\right] $$

with ${q}_{\alpha}\left({\widehat{S}}_i(t)\right)= \exp \left(-{\widehat{H}}_i(t)+{z}_{\alpha}\sqrt{\widehat{\mathrm{var}}\left({\widehat{H}}_i(t)\right)}\right).$ This can be directly implemented via the R-package survival [23]. In the case that penalized coefficients are taken without re-estimation, a practical solution to estimate their standard errors can be to compute the Hessian matrix based on the estimated parameter values.

The non-parametric bootstrap approach consists in generating *B* bootstrap samples of the original data (*B* = 200 in this work) and in estimating the model (1) for each of them. Thus, *B* models *ĥ*_1_(*t*, *T*, ***X***), …, *ĥ*_*B*_(*t*, *T*, ***X***) are obtained, allowing to estimate *B* times the expected survival probability for each patient *i* at time *t*, noted *Ŝ*_*i*,boot_(*t*) = {*Ŝ*_*i*,1_(*t*), …, *Ŝ*_*i*,*B*_(*t*)}. Then, the non-parametric confidence interval of the *Ŝ*_*i*_(*t*) based on the empirical percentiles *q*_*α*_(⋅) of the distribution of *Ŝ*_*i*, boot_(*t*) is given by

$$ {\mathrm{CI}}_{1-\theta}\left({\widehat{S}}_i(t)\right)=\left[{q}_{\frac{\theta}{2}}\left({\widehat{S}}_{i,\ \mathrm{boot}}(t)\right);\ {q}_{1-\frac{\theta}{2}}\left({\widehat{S}}_{i,\ \mathrm{boot}}(t)\right)\right]. $$

Of note, both the analytical and bootstrap confidence intervals are based on the model (1) which is estimated using a penalty parameter determined via a single cross-validation (1CV).

### Graphical visualization

**Fig. 2:**
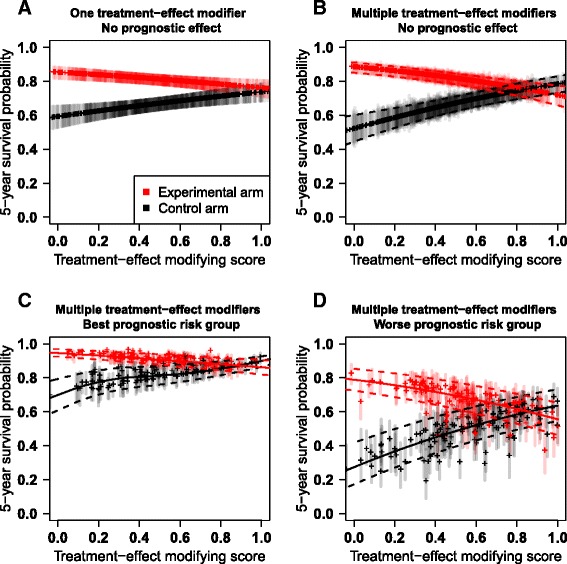
Graphical illustration of the expected survival probability at a given timepoint against the treatment-effect modifying ore. Expected survival probability against the treatment-effect modifying score $\widehat{\eta}$ in the setting of: no prognostic biomarker identified and one (**a**) or multiple (**b**) treatment-effect modifiers; multiple prognostic markers and treatment-modifiers in the lowest (**c**) and higher (**d**) prognostic risk group. Dot: point estimate, vertical line: 95% pointwise confidence interval, solid curves: average smoothed splines for point estimates, dashed curves: average smoothed splines for confidence bounds. Graphical illustrations are coming from several selected models based on a simulated dataset from the scenario 6

We visualize the expected survival probability of the patients according to their treatment-effect modifying score ${\widehat{\eta}}_i = {\displaystyle \sum_{j=1}^p}{\widehat{\gamma}}_j{X}_{i j}$ at a given horizon τ. A toy example is used for illustration in Fig. 2.

For the graphical visualization, the scores $\widehat{\boldsymbol{\eta}}=\left({\widehat{\eta}}_1,\dots, {\widehat{\eta}}_n\right)$ are scaled so that the 2.5% (i.e. ${q}_{0.025}\left(\widehat{\boldsymbol{\eta}}\right)$) and 97.5% (i.e. ${q}_{0.975}\left(\widehat{\boldsymbol{\eta}}\right)$) quantiles equal 0 and 1 respectively in the training set:

$$ {\widehat{\eta}}_i=\frac{{\widehat{\eta}}_i - {q}_{0.025}\left(\widehat{\eta}\right)}{q_{0.975}\left(\widehat{\eta}\right)-{q}_{0.025}\left(\widehat{\eta}\right)}\ . $$

In the simplest case in which only one treatment-effect modifier and no prognostic biomarker (${\widehat{\beta}}_j=0,\ \forall j$) are included in the model, the relationship between ${\widehat{\eta}}_i$ and *Ŝ*_*i*_(*t*) (and its confidence interval bounds) is strictly monotone (Fig. 2a). However, in the setting of many treatment-effect modifiers and no prognostic biomarker, two patients with equal scores ${\widehat{\eta}}_i$ have the same *Ŝ*_*i*_(*t*), but their confidence intervals may be different (Fig. 2b), due to different biomarker values. In order to obtain smoothed average confidence bounds, we considered constrained basis splines (B-splines) *spl*(*t*) [24]. Splines are numerical functions that can be used for curve-fitting by approximately fitting the data at particular nodes. The number of nodes was estimated through the Akaike Information Criterion (AIC, default criterion in the chosen R-package cobs [25]). We also forced the constraint that 0 ≤ *spl*(*t*) ≤ 1.

When the model contains also prognostic biomarkers, patients with the same treatment-effect modifying score ${\widehat{\eta}}_i$ may have different survival probability *Ŝ*_*i*_(*t*) due to different prognostic scores ${\widehat{\varphi}}_i = {\displaystyle \sum_{j=1}^p}{\widehat{\beta}}_j{X}_{i j}.$ Due to the possible large heterogeneity of *Ŝ*_*i*_(*t*) for equal scores ${\widehat{\eta}}_i$, we stratified the treatment-effect modifying plot into four groups according to the prognostic score, using the percentiles proposed by Cox [26]: 16.4%, 33.6%, 33.6% and 16.4%. For our toy example, Fig. 2c and 2d represent the relationship between *Ŝ*_*i*_(*t*) and ${\widehat{\eta}}_i$ for the best and worst prognostic risk groups, respectively. Heterogeneity within groups is still present for both point estimates of the expected survival probability and confidence interval bounds; therefore we also considered smoothing the point estimates through B-splines. Even though categorization reduces the available information [27], it leads to a more meaningful graphical representation of the prediction model.

In the rest of the study, we investigated both the *spline* strategy with prognostic categorization and the *pointwise* strategy.

## Simulation study

In this section, we present a simulation study covering several null (i.e. with no treatment-effect modifier) and alternative (i.e. with at least one true treatment-effect modifier) scenarios to evaluate the operating characteristics of the different approaches.

### Data generation and scenarios

For each simulated data set, we generated *p* = 500 Gaussian unit-variance variables, including prognostic biomarkers and/or treatment-effect modifiers. Data were generated for a total of *n* = 1500 patients, randomly assigned to the experimental or control group with equal probability of 0.5. We generated random survival times *t* with constant hazard. Table 1 shows the three null and three alternative scenarios considered in the simulations. Simulation parameters such as the desired baseline (i.e. **x** = (0, …, 0)^T^) survival probability *S*_0_(⋅) at the horizon *τ* (5 years in our simulations) and the regression parameters *α*, ***β*** and ***γ*** were chosen on the basis of the early breast cancer trial presented in the section “Application”. Thus, we fixed *S*_0_(5) = 77%. All the scenarios included censoring and an autoregressive correlation structure [28] between biomarkers $\left({\rho}_{j{ j}^{\hbox{'}}}={0.8}^{\left| j-{j}^{\hbox{'}}\right|}\right)$ within 25-biomarker blocks. As sensitivity analyses, we also investigated other values for: the baseline survival probability *S*_0_(5) (i.e. 50%), the number of biomarkers *p* (i.e. 100 and 1000) and the empirical censoring rate (i.e. between 36% and 62%) to assess their impact on the results. For each scenario, 250 data sets were generated and 250 additional ones were generated for external validation, each with the same parameters as each of the training data sets.

**Table 1:** Simulation scenarios

Scenarios	Effect size			Censoring rate	
	***α***	***β***_***j***_	***γ***_***j***_	T^−^	T^+^
(1) Complete null	0	0	0	0.72	0.72
(2) Treatment effect only	− 0.8	0	0	0.62	0.80
(3) 20 prognostic markers	0	~ *U*(−0.05, − 0.20)	0	0.70	0.70
(4) 15 treatment-effect modifiers	0	0	~ *U*(−0.10, − 0.40)	0.71	0.71
(5) Treatment effect + (4)	− 0.8	0	~ *U*(−0.10, − 0.40)	0.61	0.78
(6) 20 prognostic markers + (5)	− 0.8	~ *U*(−0.05, − 0.20)	~ *U*(−0.10, − 0.40)	0.60	0.76

T^+^: experimental arm, T^−^: control arm

### Evaluation criteria

All the evaluation criteria have been measured at *τ* = 5 years.

**Integrated brier score.** As a measure of overall prediction error of the models, we used the integrated Brier score (iBrier score, [29]). The time-dependent Brier score is a quadratic score based on the predicted time-dependent survival probability defined by

$$ \mathrm{Brier}\left(\tau \right) = \frac{1}{n}{\displaystyle \sum_{i=1}^n}\left[\frac{{\left({\widehat{S}}_C\left({t}_i\right)\right)}^2\mathbf{I}\left({t}_i\le \tau, {\delta}_i=1\ \right)}{{\widehat{S}}_C\left({t}_i\right)}+\frac{{\left(1-{\widehat{S}}_i(t)\right)}^2\mathbf{I}\left({t}_i>\tau \right)}{{\widehat{S}}_C\left(\tau \right)}\right], $$

and takes censoring into account through *Ŝ*_*C*_(*t*_*i*_) = P(*C* > *t*_*i*_), the Kaplan-Meier estimate of the censoring distribution with *t*_*i*_ the survival time of patient *i*. The integration of the Brier score can be done by over time *t* ∈ [0, *τ*] with respect to some weight function *W*(*t*) for which a natural choice is (1 − *Ŝ*(*t*))/(1 − *Ŝ*(*τ*)) [29]. The lower the iBrier score, the larger the prediction accuracy is.

**Uno’s C-statistic.** To evaluate the discrimination of the prediction model, we also measured the concordance between ${\widehat{\pi}}_i$, the linear predictor of (1), and the survival time based on the Uno’s C-statistic, one of the least biased concordance statistic estimator in the presence of censoring [30]. For the entire model, we computed

$$ \mathrm{C}\left(\tau \right)= UnoC\left(\widehat{\boldsymbol{\pi}},\tau \right) = \frac{{\displaystyle {\sum}_{i,{i}^{\hbox{'}}}}{\left({\widehat{S}}_C\left({t}_i\right)\right)}^{-2}\mathbf{I}\left({t}_i<{t}_{i^{\hbox{'}}},{t}_i<\tau \right)\ \mathbf{I}\left({\widehat{\pi}}_i>{\widehat{\pi}}_{i^{\hbox{'}}}\right){\delta}_i}{{\displaystyle {\sum}_{i,{i}^{\hbox{'}}}}{\left({\widehat{S}}_C\left({t}_i\right)\right)}^{-2}\mathbf{I}\left({t}_i<{t}_{i^{\hbox{'}}},\ {t}_i<\tau \right){\delta}_i}. $$

The larger the C(*τ*), the higher the overall discrimination is. **ΔUno’s C-statistic.** For the treatment-effect modifying component ${\widehat{\eta}}_i$, we computed the absolute difference of the arm-specific Uno’s C-statistics as we proposed previously [10] to evaluate the biomarker-by-treatment interaction strength:

$$ \Delta \mathrm{C}\left(\tau \right)=\left| UnoC\left(\widehat{\boldsymbol{\eta}},\ \tau;\ \boldsymbol{T}=+0.5\right)- UnoC\left(\widehat{\boldsymbol{\eta}},\ \tau;\ \boldsymbol{T} = -0.5\right)\right| $$

where $UnoC\left(\widehat{\boldsymbol{\eta}},\ \tau; \boldsymbol{T}= T\right)$ is the Uno’s C-statistic computed within the arm *T* only. The larger the ΔC(*τ*), the higher the interaction strength is.

As a comparator, we also computed these prediction accuracy metrics (iBrier, Uno and ΔUno) for the “oracle model” that is the unpenalized Cox proportional hazards model fitted to the truly related biomarkers in the training set and applied to the validation set.

### Bias and precision of the expected survival probability

To assess reliability of the estimation of the expected survival probability, we compared for each patient *i*, its estimated survival probability *Ŝ*_*i*_(*τ*) to its theoretical survival probability *S*_*i*_(*τ*). The theoretical survival probability is computed from the baseline survival and the regression parameters of the simulation model. We evaluated the accuracy of *Ŝ*_*i*_(*τ*) through the mean bias

$$ \mathrm{Mean}\ \mathrm{Bias}\ \left(\mathrm{MB}\right) = \frac{1}{n}{\displaystyle \sum_{i=1}^n}\left({\widehat{S}}_i\left(\tau \right) - {S}_i\left(\tau \right)\right) $$

and its precision through its standard error

$$ \mathrm{Standard}\ \mathrm{E}\mathrm{rror}\ \left(\mathrm{SE}\right)=\sqrt{\frac{1}{n-1}{\displaystyle \sum_{i=1}^{\mathrm{n}}}{\left({\widehat{S}}_i\left(\tau \right) - \mathrm{E}\left({\widehat{S}}_i\left(\tau \right)\right)\right)}^2}. $$

Finally, to evaluate the accuracy of the developed confidence intervals, we also evaluated their empirical coverage probability as

$$ \mathrm{Coverage}\ \mathrm{Probability}\ \left(\mathrm{CP}\right)=\frac{1}{n}{\displaystyle \sum_{i=1}^n}\mathbf{I}\left({q}_{\frac{\theta}{2}}\left({\widehat{S}}_i\left(\tau \right)\right)\le {S}_i\left(\tau \right)\le {q}_{1-\frac{\theta}{2}}\left({\widehat{S}}_i\left(\tau \right)\right)\right). $$

## Results

The results of the simulation study are summarized in Tables 2 and 3. These tables summarize both the predictive accuracy metrics and the accuracy and precision of survival estimates of the time-to-event regression models subject to the adaptive lasso penalty. As the lasso penalty provided slightly worse coverage of the confidence intervals and obtained slightly less good biomarker-selection performance in some scenarios [10], its results are given in the Additional files 1 and 2.

**Table 2:** Prediction measures of the selected models by the adaptive lasso penalty

Scenarios	Integrated Brier score (iBrier)				Uno’s C-statistic (C)				Δ Uno’s C-statistic (ΔC)			
	Training		Validation		Training		Validation		Training		Validation	
	1cv	2cv	Selected model	Oracle model	1cv	2cv	Selected model	Oracle model	1cv	2cv	Selected model	Oracle model
(1) Complete null	0.094	0.099	0.099	0.098	0.636	0.497	0.499	0.500	0.070	0.030	0.002	0.000
(2) Treatment effect only	0.096	0.102	0.101	0.100	0.663	0.586	0.586	0.558	0.062	−0.001	−0.001	0.000
(3) 20 prognostic markers	0.097	0.105	0.105	0.102	0.717	0.630	0.630	0.665	0.062	−0.003	0.000	0.000
(4) 15 treatment-effect modifiers	0.094	0.106	0.105	0.101	0.726	0.570	0.571	0.641	0.334	0.209	0.229	0.283
(5) Treatment effect + (4)	0.094	0.107	0.106	0.102	0.740	0.621	0.621	0.675	0.332	0.206	0.225	0.284
(6) 20 prognostic markers + (5)	0.096	0.111	0.109	0.104	0.767	0.669	0.670	0.718	0.296	0.183	0.207	0.266

1cv and 2cv: single and double cross-validation in the training set. The selected model is the penalized model obtained by single cross-validation in the training set (1cv) and applied to the validation set. The oracle model is the unpenalized Cox proportional hazards model fitted to the truly related biomarkers in the training set and applied to the validation set. Average quantities across 250 replications

**Table 3:** Accuracy and precision of the survival probabilities, and coverage probability of their 95% confidence intervals of the selected models by the adaptive lasso penalty

Scenarios	Point estimate of the 5-year survival probability				95% CI of the expected survival			
	Mean bias		Standard error		Coverage probability			
	Pointwise	Spline	Pointwise	Spline	Pointwise		Spline	
					Anly	Boot	Anly	Boot
(1) Complete null	−0.002	−0.001	0.05	0.05	0.93	0.97	0.94	1.00
(2) Treatment effect only	−0.001	−0.001	0.06	0.05	0.93	0.96	0.93	1.00
(3) 20 prognostic biomarkers	−0.002	0.001	0.09	0.09	0.91	0.97	0.89	0.98
(4) 15 treatment-effect modifiers	−0.003	−0.001	0.11	0.10	0.88	0.96	0.89	0.98
(5) Treatment effect + (4)	−0.002	0.000	0.11	0.10	0.88	0.96	0.89	0.98
(6) 20 prognostic biomarkers + (5)	−0.005	0.000	0.13	0.13	0.88	0.96	0.87	0.96

Anly: analytical approach, Boot: non-parametric bootstrap approach, CI: confidence interval. Average quantities across 250 replications

The models identified through the adaptive lasso penalty provided predictive accuracy (Table 2) relatively close to the oracle models. Computed on an external validation data set, the iBrier score was quite low (varying from 0.099 to 0.109) and the Uno’s C-statistic was quite large in presence of prognostic effects (scenarios 2 and 6, varying from 0.586 to 0.670). The ΔUno’s C-statistic was also large in presence of true treatment-effect modifiers (scenarios 4–6, varying from +0.207 to +0.229). When evaluating these criteria on the training set used to compute the prediction model, we observed overrated performances of the models as compared to results in external data (training 1cv *vs.* validation: from +0.077 to +0.155 for C and from +0.062 to +0.107 for ΔC). The double cross-validation technique (training 2cv) gave internal results close to those of an external validation (validation).

When focusing on the estimation of the survival probability (Table 3), the mean bias was close to zero in all scenarios. Nonetheless, for *S*_0_ ~ 50% (Additional file 3), a slight negative bias was observed (from −0.007 to −0.020). In terms of precision, the standard error of the survival point estimate was low in absence of biomarker effects (null scenarios 1–2, from 0.05 to 0.06) and was higher in presence of prognostic biomarkers (null scenario 3, 0.09) and even higher with treatment-effect modifiers (scenarios 4–5, from 0.10 to 0.11). The variability was the largest when both prognostic and treatment-modifiers were present (scenario 6, SE from 0.13). No relevant difference in terms of accuracy and precision of survival point estimate was observed between the pointwise and spline strategy (Table 3). The standard error was inflated, too, when refitting the selected regression model in a second step (“refit adaptive lasso”) to obtain unpenalized coefficients (e.g. scenario 6: SE from 0.16 to 0.17, Additional file 4).

For the computation of the 95% confidence interval of the estimated survival probability, the non-parametric bootstrap approach outperformed the analytical approach in terms of empirical coverage (Table 3). Despite the former (through the pointwise strategy) produced slightly overly conservative confidence intervals (CP from 0.96 to 0.97), the latter was more biased and in the opposite direction (CP from 0.91 to 0.93 in null scenarios and 0.88 in alternative scenarios). These probabilities for the analytical approach were even lower when using the refit model (CP from 0.68 to 0.73, Additional file 4), whereas remained unchanged for the bootstrap approach.

The categorization of the prognostic scores into risk groups and the use of splines slightly increased the coverage probability of the confidence intervals for the bootstrap approach (increase from 0.96–0.97 to 0.96–1.00) whereas the coverage probability remained unchanged for the analytical approach.

In sensitivity analyses, the overall number of biomarkers *p* and the empirical censoring rate slightly impacted the results. Indeed, for smaller *p* and lower censoring rate, the prediction accuracy metrics were closer to the oracle models and the overfitting issue was smaller (Additional files 5 and 6). Also, the precision of the expected survival estimates was higher for this setting (Additional files 7 and 8).

## Application

**Fig. 3:**
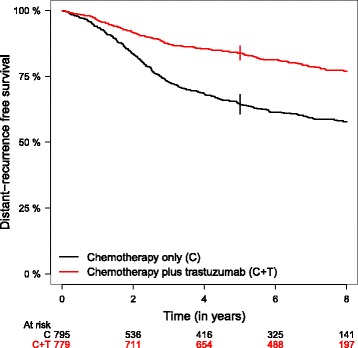
Arm-specific distant-recurrence free survival in the illustrated breast cancer trial. Vertical lines: 95% confidence interval at 5 years

A retrospective biomarker study was performed on tumor samples from *n* = 1574 patients in an early breast cancer randomized clinical trial comparing chemotherapy plus adjuvant trastuzumab (arm C + T, *n* = 779) or not (arm C, *n* = 795). The comprehensive description of this data set is provided in Pogue-Geile et al. [1]. Gene expression data had been collected for *p* = 462 genes and normalized as in the original publication. Clinicopathological covariates such as ER and nodal status, and tumor size were also available. Median follow-up time for distant-recurrence free survival (DRFS) was 7.1 years and the censoring rate was 73% (i.e. 431 events for DRFS). The arm-specific 5-year DRFS was 84% (CI95%: 81%–86%) and 64% (CI95%: 61%–68%) for patients in arm C + T and C, respectively (Fig. 3). In the 1574 patients, adjuvant trastuzumab led to significant better DRFS as compared to chemotherapy alone (Hazard Ratio = 0.46 [95% CI: 0.38–0.56]).

**Fig. 4:**
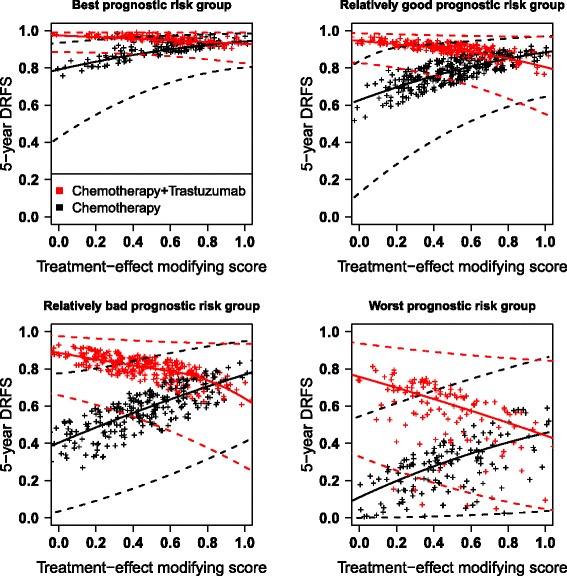
5-year distant-recurrence free survival against the treatment-effect modifying score of the effect of trastuzumab in early breast cancer. Graphical representation of the model showed in Table 4. DRFS: distant-recurrence free survival, dot: point estimate, solid curves: average smoothed splines for point estimates, dashed curves: average smoothed splines for confidence bounds

The clinico-genomic prediction model, established through the model (1) subject to the adaptive lasso penalty, is presented in Table 4. It contains 102 prognostic variables (4 clinical and 98 genomic variables) and 24 treatment-effect modifiers. Interestingly, some prognostic biomarkers have already been identified in the biomedical literature, such as SOX4 [31] or CSNK1D [32]. In addition, some immune genes were also identified in the treatment-effect modifying component: CD9 and CCL21, which is consistent with some articles highlighting the involvement of immune pathways in the efficacy of trastuzumab [33, 34]. Fig. 4 provides a graphical visualization of the model representing the 5-year DRFS according to the treatment-effect modifying score for different prognostic risk groups. Confidence intervals were estimated through the non-parametric bootstrap approach (*B* = 200) and were smoothed via B-splines using two or three nodes estimated by AIC. The prognostic score was categorized through the distribution proposed by Cox [26], i.e. group 1 ($\widehat{\boldsymbol{\varphi}}$ < 0.20), group 2 (0.20 ≤ $\widehat{\boldsymbol{\varphi}}$ < 0.99), group 3 (0.99 ≤ $\widehat{\boldsymbol{\varphi}}$ < 1.85) and group 4 (1.85 ≥ $\widehat{\boldsymbol{\varphi}}$). The entire prediction model (i.e. 126 variables) has a moderate ability to discriminate patients according to their survival probability (C = 0.67 for a double cross-validation). Regarding the treatment-effect modifying component of the model, it only slightly discriminates patients for their treatment benefit (ΔC = +0.02 for a double cross-validation): the lowest the treatment-effect modifying score, the highest the benefit of the trastuzumab is. As expected, when performing a single cross-validation the prediction measures are higher (C = 0.80 and ΔC = 0.23). In terms of point estimates, the effect of treatment with trastuzumab on absolute DRFS seems higher for the low treatment-effect modifying scores. However, the width of the confidence intervals is extremely large and the confidence intervals largely overlap between the two arms. The selected model subject to the lasso penalty identified only the SIAH2 gene as treatment-effect modifier (Additional file 9). Some recent articles have been proposed to reduce the number of selected biomarkers by selecting a more stringent tuning parameter. We previously proposed the *pcvl* criterion [35] that reduces the number of selected penalized biomarkers in the present application from 122 to 64 for the adaptive lasso (Additional file 10).

**Table 4:** Developed clinico-genomic model through the full biomarker-by-treatment interaction Cox model subject to the adaptive lasso penalty

Prognostic component	
*Clinical variables* (*p* = 4)	Treatment (−0.889^u^), ER status (−0.091^u^), Tumor size (0.175^u^), Nodal status (0.418^u^)
*Genomic variables* (*p* = 98)	ACTB (0.020), ADCYAP1 (0.009), ANGPTL4 (0.034), ARL8A (0.020), BBC3 (−0.088), BDH2 (−0.067), CAPS (0.064), CASC3 (−0.058), CCDC74A (0.080), CDC6 (−0.069), CDH3 (0.027), CFLP1 (−0.143), CSNK1A1 (−0.079), CSNK1D (−0.063), CXXC5 (−0.141), DHPS (0.148), DNAJC4 (−0.154), DPY19L4 (0.015), ELAVL4 (−0.107), ELN (0.015), ENO1 (0.012), ERBB4 (−0.047), FABP5 (0.063), FAM84B (−0.084), FBXW11 (0.069), FKSG30 (−0.049), FLJ22659 (0.006), FLJ22795 (0.009), FLJ35390 (0.096), FRAG1 (0.075), FRMD4A (0.106), GHR (−0.067), GPRIN1 (0.009), GSN (0.039), HIST1H2AA (−0.085), HIST2H2BE (0.009), IDUA (0.038), IGJ (−0.110), IGKV2.24 (0.029), ILF2 (0.014), KCNE4 (−0.075), KIAA1920 (−0.025), KIF2C (0.093), KRT81 (−0.106), L3MBTL2 (−0.057), LCE3E (−0.101), LOC400590 (−0.021), MAD2L2 (−0.098), MAP3K13 (0.115), MBOAT2 (0.101), MED13L (−0.090), METTL3 (−0.138), MSI2 (−0.039), MTCH2 (0.018), MVP (0.068), NAT1 (−0.019), NAT10 (−0.085), NDC80 (0.082), NECAB3 (0.075), NXPH3 (0.001), OGFR (−0.040), PCK2 (−0.061), PGM5 (0.139), PHGDH (0.107), PITPNC1 (0.089), PRPF40A (0.041), PTTG1 (0.091), RBM14 (0.090), RELB (−0.016), RHBDD1 (−0.070), RND3 (0.022), RPL34 (<0,001), RPS2 (−0.050), SFRP1 (−0.121), SLC25A28 (−0.057), SLC25A31 (0.154), SLC25A5 (−0.047), SLC30A10 (0.018), SLC6A19 (−0.056), SMCP (0.055), SOX4 (0.112), SPDEF (0.079), SPP1 (0.090), ST6GALNAC4 (−0.058), STEAP3 (−0.005), STK11IP (−0.009), SULT1A2 (−0.085), TBXAS1 (<0,001), TCEB2 (0.058), TFRC (−0.132), TMSB10 (−0.096), TRABD (−0.037), TUBB2C (0.103), UBE2W (0.116), UGDH (0.039), XYLT1 (0.082), ZNF592 (0.072), ZNF609 (−0.081)
Treatment-effect modifying component	
*Genomic variables* (*p* = 24)	ATAD3A (−0.100), C16orf14 (0.165), C1orf93 (−0.115), CCL21 (−0.046), CD9 (−0.191), CIAPIN1 (−0.063), CLIC1 (0.148), DKFZP434A0131 (0.167), FAM148A (−0.085), FNDC4 (0.010), FURIN (0.030), KRTAP2.4 (0.256), MED13L (0.046), MIA (−0.064), MMD (−0.104), ORMDL3 (−0.023), RPLP0 (0.006), SIAH2 (0.123), SLC39A14 (0.007), SSBP2 (−0.099), THOP1 (−0.279), THRAP1 (−0.049), TMEM45B (−0.169), UNC119 (0.017)
Prediction measures	
C-statistic (C)	0.80 (1CV), 0.67 (2CV)
ΔC-statistic (ΔC)	0.23 (1CV), 0.02 (2CV)

^u^unpenalized regression coefficient, 1CV and 2CV: single and double cross-validation

## Discussion

Individual predictions for future patients are the ultimate goal of the stratified medicine era [36]. Individual outcome predictions from population risks are most often affected by an inherently large amount of uncertainty. Nevertheless, the expected survival probabilities under different treatment alternatives can be a valuable tool to inform therapeutic decision-making.

In the present study, we proposed a unified framework for developing, evaluating and visualizing a prediction tool for future patients in the setting of randomized clinical trials with a survival endpoint in a high-dimensional space. We performed a simulation study covering null and alternative scenarios to evaluate different approaches. Based on simulations, the models identified either with the lasso or the adaptive lasso penalties give close results, with accurate and precise estimations of the expected survival probability. The lasso penalty give slightly worse results than the adaptive lasso penalty for the construction of confidence intervals through the analytical approach; however, for both penalized models, the coverage probabilities of their non-parametric 95% confidence intervals are close to the nominal level.

The simulations also highlighted that refitting the regression model after biomarker selection to obtain unpenalized coefficients reduced the precision of the expected survival probability estimations. In case no external data is available, a double cross-validation is useful to reduce the over-optimism of the measures computed internally (prediction error through the iBrier score and discrimination through the Uno’s C-statistics). Other resampling techniques such as bootstrap or jackknife could have been also proposed, but are known to be more time consuming. In addition, a previous study comparing resampling methods highlighted that the cross-validation performed well for predictive accuracy [37]. Regarding the construction of confidence intervals, the non-parametric (bootstrap) confidence intervals outperform the analytical approach in our simulation study. A possible explanation is that standard errors of penalized regression coefficients are difficult to estimate, we used the Hessian matrix based on the estimated parameter values in this study. Furthermore, this approach does not take into account the upstream variable selection process, which adds variability and could explain why the coverage probability is lower than the nominal level.

Finally, we also considered to possibly use smoothing splines and to categorize the prognostic score into four risk groups to obtain a more meaningful graphical visualization of the model. Of course, the cut-offs are arbitrary and should be predefined by warranting a sufficient number of patients per group. Even if the categorization of the prognostic scores slightly reduces the information, this is counterbalanced by a more straightforward graphical representation of the model predictions. In particular, this reduction in coverage probability is minimal with the bootstrap approach.

To the best of our knowledge, no simulation study has previously investigated the accuracy and precision of expected survival probabilities from a penalized Cox regression model investigating in high-dimensional spaces both prognostic biomarkers and treatment-effect modifiers in a randomized clinical trial. Matsui et al. [3] showed in a real data example how to obtain individual estimations from prediction models based on both prognostic biomarkers and treatment-effect modifiers. However, they did not mention the problem of computing confidence intervals and selected the biomarkers through a univariate approach, which is suboptimal in a high-dimensional setting [10]. The inference of survival probabilities is a topic increasingly discussed in the literature. Two recent articles [5, 6] focused on a prognostic setting, mainly in low-dimensional spaces. Sinnott and Cai [5] proposed a two-step ensemble voting approach and Lin and Halabi [6] proposed a perturbation approach to better estimate the standard errors, but did not investigate the impact on expected survival probabilities.

We also applied the proposed strategies to a randomized clinical trial of early breast cancer. Interestingly, the identified model contains some biomarkers already known in the biomedical literature but the interaction strength seemed low based on double cross-validation. Due to the low number of events of the complete data set, we did not separate it into training and validation sets. Of note, the large width of the confidence intervals of the survival predictions translates the high uncertainty around these measures, as also discussed by [5]. The identified model contains a very large number of biomarkers, but parsimonious models could be preferred. Some strategies have been proposed to reduce the number of selected biomarkers by selecting a more stringent tuning parameter such as the *pcvl* criterion [35]. Finally, a remaining open question concerns the integration of clinical and genomic variables. Ongoing research focuses on this topic, but no consensus has been reached yet [38, 39]. In the application shown, we left the clinical variables unpenalized because they are known to have a strong prognostic main effect in the early breast cancer setting.

## Conclusions

In this paper, we propose a unified framework for developing and validating a prediction model with treatment-effect modifiers from high-dimensional survival data in a randomized clinical trial. We suggest to: internally estimate the performance of the model through a double cross-validation; estimate the expected survival probabilities at a given horizon for future patients and construct confidence intervals thereof using bootstrap; and visualize them using different plots for different prognostic risk groups with smoothed splines to obtain a meaningful graphical visualization.

## Additional files

Additional file 1: Prediction measures of the selected models by the lasso penalty. Additional results of the simulation study. (DOCX 16 kb)

Additional file 2: Accuracy and precision of the survival probabilities, and coverage probability of their 95% confidence intervals of the selected models by the lasso penalty. Additional results of the simulation study. (DOCX 16 kb)

Additional file 3: Accuracy and precision of the survival probabilities, and coverage probability of the associated 95% confidence intervals of the selected models by the adaptive lasso penalty (scenarios with *S*_0_ ~ 50%). Additional results of the simulation study. (DOCX 17 kb)

Additional file 4: Accuracy and precision of the survival probabilities, and coverage probability of the associated 95% confidence intervals of the selected models by the refit adaptive lasso penalty. Additional results of the simulation study. (DOCX 16 kb)

Additional file 5: Prediction measures of the selected models by the adaptive lasso penalty (scenarios with *p* = 100 and 1000). Additional results of the simulation study. (DOCX 21 kb)

Additional file 6: Prediction measures of the selected models by the adaptive lasso penalty (scenarios with lower censoring rate). Additional results of the simulation study. (DOCX 18 kb)

Additional file 7: Accuracy and precision of the survival probabilities, and coverage probability of the associated 95% confidence intervals of the selected models by the adaptive lasso penalty (scenarios with *p* = 100 and 1000). Additional results of the simulation study. (DOCX 18 kb)

Additional file 8: Accuracy and precision of the survival probabilities, and coverage probability of the associated 95% confidence intervals of the selected models by the adaptive lasso penalty (scenarios with lower censoring rate). Additional results of the simulation study. (DOCX 18 kb)

Additional file 9: Developed clinico-genomic model through the full biomarker-by-treatment interaction Cox model subject to the lasso penalty. Additional results of the breast cancer application. (DOCX 15 kb)

Additional file 10: Developed clinico-genomic model through the full biomarker-by-treatment interaction Cox model subject to the adaptive lasso penalty with λ selected through the *pcvl* criterion. Additional results of the breast cancer application. (DOCX 15 kb)

## Abbreviations

Anly: Analytical approach
Boot: Bootstrap approach
B-splines: Basis splines
C: Uno’s C-statistic
CI: Confidence interval
CP: Coverage probability
CV: Cross-validation
DRFS: Distant-recurrence free survival
iBrier: Integrated Brier score
MB: Mean bias
SE: Standard error
ΔC: Difference in Uno’s C-statistic between the two treatment arms
