# Supplementary material for: Robust estimation of the expected survival probabilities from high-dimensional Cox models with biomarker-by-treatment interactions in randomized clinical trials
Source: BMC Med Res Methodol. 2017 May 22;17:83. doi: 10.1186/s12874-017-0354-0 (PMC5441049; doi:10.1186/s12874-017-0354-0)
Supplement: Supplementary file 1 — Prediction measures of the selected models by the lasso penalty. Additional results of the simulation study. (DOCX 16 kb) [file 12874_2017_354_MOESM1_ESM.docx]

**ADDITIONAL FILE 1:** Prediction measures of the selected models by the lasso penalty

| **Scenarios** | **Integrated Brier score (iBrier)** | | | | **Uno’s C-statistic (C)** | | | | **Δ Uno’s C-statistic (ΔC)** | | | |
| --- | --- | --- | --- | --- | --- | --- | --- | --- | --- | --- | --- | --- |
|  | Training | | Validation | | Training | | Validation | | Training | | Validation | |
|  | 1cv | 2cv | Selected model | Oracle model | 1cv | 2cv | Selected model | Oracle model | 1cv | 2cv | Selected model | Oracle model |
| (**1**) Complete null | 0.097 | 0.098 | 0.098 | 0.098 | 0.538 | 0.495 | 0.498 | 0.500 | 0.002 | 0.000 | 0.000 | 0.000 |
| (**2**) Treatment effect only | 0.099 | 0.100 | 0.100 | 0.100 | 0.588 | 0.583 | 0.575 | 0.557 | 0.004 | 0.000 | 0.000 | 0.000 |
| (**3**) 20 prognostic markers | 0.098 | 0.104 | 0.104 | 0.102 | 0.717 | 0.632 | 0.640 | 0.665 | 0.087 | -0.005 | 0.000 | 0.000 |
| (**4**) 15 treatment-effect modifiers | 0.098 | 0.105 | 0.104 | 0.101 | 0.681 | 0.545 | 0.568 | 0.641 | 0.265 | 0.138 | 0.188 | 0.283 |
| (**5**) Treatment effect + (4) | 0.099 | 0.106 | 0.105 | 0.102 | 0.691 | 0.612 | 0.621 | 0.675 | 0.253 | 0.137 | 0.178 | 0.284 |
| (**6**) 20 prognostic markers + (5) | 0.097 | 0.110 | 0.108 | 0.104 | 0.766 | 0.669 | 0.680 | 0.718 | 0.303 | 0.180 | 0.209 | 0.266 |
| 1cv and 2cv: single and double cross-validation in the training set. The selected model is the penalized model obtained by single cross-validation in the training set (1cv) and applied to the validation set. The oracle model is the unpenalized Cox proportional hazards model fitted to the truly related biomarkers in the training set and applied to the validation set. Average quantities across 250 replications. | | | | | | | | | | | | |
