# Supplementary material for: Robust estimation of the expected survival probabilities from high-dimensional Cox models with biomarker-by-treatment interactions in randomized clinical trials
Source: BMC Med Res Methodol. 2017 May 22;17:83. doi: 10.1186/s12874-017-0354-0 (PMC5441049; doi:10.1186/s12874-017-0354-0)
Supplement: Supplementary file 3 — Accuracy and precision of the survival probabilities, and coverage probability of the associated 95% confidence intervals of the selected models by the adaptive lasso penalty (scenarios with S 0 ~ 50%). Additional results of the simulation study. (DOCX 17 kb) [file 12874_2017_354_MOESM3_ESM.docx]

**ADDITIONAL FILE 3:** Accuracy and precision of the survival probabilities, and coverage probability of the associated 95% confidence intervals of the selected models by the adaptive lasso penalty (scenarios with$S_{0}\sim50\%$)

| **Scenarios** | **Point estimate of the 5-year survival probability** | | | |  | **95% CI of the expected survival** | | | |
| --- | --- | --- | --- | --- | --- | --- | --- | --- | --- |
|  | Mean bias | | Standard error | |  | Coverage probability | | | |
|  | Pointwise | Spline | Pointwise | Spline |  | Pointwise | | Spline | |
|  |  |  |  |  |  | Anly | Boot | Anly | Boot |
| (**1**) Complete null | -0.007 | -0.007 | 0.09 | 0.09 |  | 0.94 | 0.98 | 0.95 | 1.00 |
| (**2**) Treatment effect only | -0.009 | -0.008 | 0.08 | 0.08 |  | 0.95 | 0.98 | 0.96 | 1.00 |
| (**3**) 20 prognostic biomarkers | -0.013 | -0.012 | 0.12 | 0.13 |  | 0.92 | 0.97 | 0.89 | 0.99 |
| (**4**) 15 treatment-effect modifiers | -0.020 | -0.020 | 0.16 | 0.15 |  | 0.91 | 0.97 | 0.92 | 0.99 |
| (**5**) Treatment effect + (4) | -0.017 | -0.017 | 0.16 | 0.15 |  | 0.90 | 0.97 | 0.91 | 0.98 |
| (**6**) 20 prognostic biomarkers + (5) | -0.019 | -0.019 | 0.16 | 0.16 |  | 0.90 | 0.96 | 0.89 | 0.96 |
| Anly: analytical approach, Boot: non-parametric bootstrap approach, CI: confidence interval. Average quantities across 250 replications. | | | | | | | | | |
