# Supplementary material for: Robust estimation of the expected survival probabilities from high-dimensional Cox models with biomarker-by-treatment interactions in randomized clinical trials
Source: BMC Med Res Methodol. 2017 May 22;17:83. doi: 10.1186/s12874-017-0354-0 (PMC5441049; doi:10.1186/s12874-017-0354-0)
Supplement: Supplementary file 7 — Accuracy and precision of the survival probabilities, and coverage probability of the associated 95% confidence intervals of the selected models by the adaptive lasso penalty (scenarios with p = 100 and 1000). Additional results of the simulation study. (DOCX 18 kb) [file 12874_2017_354_MOESM7_ESM.docx]

**ADDITIONAL FILE 7:** Accuracy and precision of the survival probabilities, and coverage probability of the associated 95% confidence intervals of the selected models by the adaptive lasso penalty (scenarios with *p* = 100 and 1000)

|  |  | **Point estimate of the 5-year survival probability** | | | |  | **95% CI of the expected survival** | | | | |
| --- | --- | --- | --- | --- | --- | --- | --- | --- | --- | --- | --- |
|  |  | Mean bias | | Standard error | |  | Coverage probability | | | | |
|  |  | Pointwise | Spline | Pointwise | Spline |  | Pointwise | | | Spline | |
|  |  |  |  |  |  |  | Anly | | Boot | Anly | Boot |
| *p* = 100 biomarkers | (**1**) Complete null | -0.001 | 0.000 | 0.03 | 0.03 |  | 0.92 | | 0.97 | 0.93 | 1.00 |
|  | (**2**) Treatment effect only | 0.000 | 0.000 | 0.03 | 0.03 |  | 0.93 | | 0.97 | 0.95 | 1.00 |
|  | (**3**) 20 prognostic biomarkers | -0.002 | 0.001 | 0.07 | 0.08 |  | 0.91 | | 0.96 | 0.85 | 0.95 |
|  | (**4**) 15 treatment-effect modifiers | -0.002 | -0.001 | 0.08 | 0.07 |  | 0.90 | | 0.96 | 0.90 | 0.97 |
|  | (**5**) Treatment effect + (4) | -0.001 | -0.001 | 0.08 | 0.07 |  | 0.89 | | 0.96 | 0.90 | 0.97 |
|  | (**6**) 20 prognostic biomarkers + (5) | -0.003 | 0.002 | 0.10 | 0.10 |  | 0.89 | | 0.95 | 0.86 | 0.92 |
| *p* = 1000 biomarkers | (**1**) Complete null | -0.001 | 0.001 | 0.07 | 0.06 |  | | 0.92 | 0.96 | 0.92 | 1.00 |
|  | (**2**) Treatment effect only | -0.002 | -0.001 | 0.07 | 0.07 |  | | 0.92 | 0.96 | 0.92 | 1.00 |
|  | (**3**) 20 prognostic biomarkers | -0.004 | 0.000 | 0.10 | 0.10 |  | | 0.90 | 0.97 | 0.89 | 0.98 |
|  | (**4**) 15 treatment-effect modifiers | -0.006 | -0.003 | 0.13 | 0.12 |  | | 0.87 | 0.96 | 0.88 | 0.98 |
|  | (**5**) Treatment effect + (4) | -0.005 | -0.002 | 0.13 | 0.12 |  | | 0.87 | 0.96 | 0.88 | 0.97 |
|  | (**6**) 20 prognostic biomarkers + (5) | -0.008 | -0.003 | 0.14 | 0.14 |  | | 0.88 | 0.96 | 0.87 | 0.96 |
| Anly: analytical approach, Boot: non-parametric bootstrap approach, CI: confidence interval. Average quantities across 250 replications. | | | | | | | | | | | |
