# Supplementary material for: Robust estimation of the expected survival probabilities from high-dimensional Cox models with biomarker-by-treatment interactions in randomized clinical trials
Source: BMC Med Res Methodol. 2017 May 22;17:83. doi: 10.1186/s12874-017-0354-0 (PMC5441049; doi:10.1186/s12874-017-0354-0)
Supplement: Supplementary file 9 — Developed clinico-genomic model through the full biomarker-by-treatment interaction Cox model subject to the lasso penalty. Additional results of the breast cancer application. (DOCX 15 kb) [file 12874_2017_354_MOESM9_ESM.docx]

**ADDITIONAL FILE 9:** Developed clinico-genomic model through the full biomarker-by-treatment interaction Cox model subject to the lasso penalty

| **Prognostic component** | |
| --- | --- |
| *Clinical variables*  (*p*=4) | Treatment (-0.852^u^), ER status (-0.182^u^), Tumor size (0.128^u^), Nodal status (0.422^u^) |
| *Genomic variables*  **(***p*=27) | ACTB (0.003), ANLN (0.002), ARL8A (0.003), CASC3 (-0.001), CFLP1 (-0.052),  CSNK1D (-0.007), CXXC5 (-0.017), DNAJC4 (-0.020), FABP5 (0.040), GHR (-0.009), HIST1H2AA (-0.026), IGH. (-0.023), IGJ (-0.067), ILF2 (0.009), KIF2C (<0,001),  MED13L (-0.055), METTL3 (-0.056), NDC80 (0.001), PKP3 (0.003), RPS2 (-0.041),  SOX4 (0.047), SPP1 (0.070), ST6GALNAC4 (-0.006), SULT1A2 (-0.013),  TRABD (-0.049), TUBB2C (0.026), XYLT1 (0.010) |
| **Treatment-effect modifying component** | |
| *Genomic variables*  (*p*=1) | SIAH2 (0.009) |
| **Prediction measures** | |
| C-statistic (C) | 0.72 (1CV), 0.67 (2CV) |
| ΔC-statistic (ΔC) | 0.06 (1CV), 0.02 (2CV) |
| ^u^ unpenalized regression coefficient, 1CV and 2CV: single and double cross-validation. | |
