# Supplementary material for: Robust estimation of the expected survival probabilities from high-dimensional Cox models with biomarker-by-treatment interactions in randomized clinical trials
Source: BMC Med Res Methodol. 2017 May 22;17:83. doi: 10.1186/s12874-017-0354-0 (PMC5441049; doi:10.1186/s12874-017-0354-0)
Supplement: Supplementary file 10 — Developed clinico-genomic model through the full biomarker-by-treatment interaction Cox model subject to the adaptive lasso penalty with λ selected through the pcvl criterion. Additional results of the breast cancer application. (DOCX 15 kb) [file 12874_2017_354_MOESM10_ESM.docx]

**ADDITIONAL FILE 10:** Developed clinico-genomic model through the full biomarker-by-treatment interaction Cox model subject to the adaptive lasso penalty with λ selected through the *pcvl* criterion

| **Prognostic component** | |
| --- | --- |
| *Clinical variables*  (*p*=4) | Treatment (-0.864^u^), ER status (-0.122^u^), Tumor size (0.148^u^), Nodal status (0.429^u^) |
| *Genomic variables*  (*p*=55) | AGPS (0.008), ARL8A (0.006), CAPS (0.043), CASC3 (-0.029), CCDC74A (0.039),  CDC6 (-0.015), CFLP1 (-0.130), CSNK1A1 (-0.011), CSNK1D (-0.045), CXXC5 (-0.071), DHPS (0.111), DNAJC4 (-0.070), ELAVL4 (-0.065), FABP5 (0.054), FKSG30 (-0.020), FLJ22795 (0.009), FLJ35390 (0.010), FRAG1 (0.010), FRMD4A (0.035), GHR (-0.042), HIST1H2AA (-0.070), IGH. (-0.010), IGJ (-0.109), ILF2 (0.011), KCNE4 (-0.023),  KIF2C (0.080), KRT81 (-0.040), L3MBTL2 (-0.015), MAD2L2 (-0.059), MAP3K13 (0.037), MBOAT2 (0.036), MED13L (-0.098), METTL3 (-0.114), NAT10 (-0.032), NDC80 (0.066), OGFR (-0.015), PCK2 (-0.012), PGM5 (0.089), PITPNC1 (0.017), RPS2 (-0.079),  SFRP1 (-0.016), SLC25A28 (<0.001), SLC25A31 (0.111), SLC25A5 (-0.009),  SOX4 (0.071), SPP1 (0.070), ST6GALNAC4 (-0.018), SULT1A2 (-0.047), TCEB2 (0.049), TFRC (-0.035), TMSB10 (-0.001), TRABD (-0.056), TUBB2C (0.098), XYLT1 (0.084), ZNF609 (-0.035) |
| **Treatment-effect modifying component** | |
| *Genomic variables*  (*p*=9) | C16orf14 (0.049), CD9 (-0.052), DKFZP434A0131 (0.043), FAM148A (-0.037),  KRTAP2.4 (0.115), ORMDL3 (-0.005), SIAH2 (0.148), THOP1 (-0.156),  TMEM45B (-0.039) |
| **Prediction measures** | |
| C-statistic (C) | 0.76 (1CV), 0.67 (2CV) |
| ΔC-statistic (ΔC) | 0.14 (1CV), 0.03 (2CV) |
| ^u^ unpenalized regression coefficient, 1CV and 2CV: single and double cross-validation. | |
